# Post-COVID central hypersomnia, a treatable trait in long COVID: 4 case reports

**DOI:** 10.3389/fneur.2024.1349486

**Published:** 2024-02-14

**Authors:** Clémence Morelli-Zaher, Andrea Vremaroiu-Coman, Nicolas Coquoz, Léon Genecand, Marco Altarelli, Alzbeta Binkova, Isabelle Frésard, Pierre-Olivier Bridevaux, Grégoire Gex

**Affiliations:** ^1^Division of Pulmonology, Centre Hospitalier du Valais Romand, Hôpital du Valais, Sion, Switzerland; ^2^Division of Pulmonology, Geneva University Hospitals, Geneva, Switzerland; ^3^Faculty of Medicine, University of Geneva, Geneva, Switzerland

**Keywords:** idiopathic hypersomnia, central hypersomnia, narcolepsy, long COVID, post-COVID condition, treatment, methylphenidate, SARS-CoV-2

## Abstract

**Introduction:**

Fatigue is the most commonly reported post-COVID symptom. A minority of patients also report excessive daytime sleepiness, which could be a target for treatment.

**Methods:**

Among 530 patients with a post-COVID condition, those with excessive daytime sleepiness were systematically assessed for objective central hypersomnia, with exclusion of all cases not clearly attributable to SARS-CoV-2 infection.

**Results:**

Four cases of post-COVID central hypersomnia were identified, three fulfilling the criteria of the 3rd International Classification of Sleep Disorders for idiopathic hypersomnia, and one for type II narcolepsy. We report here their clinical history, sleep examination data and treatment, with a favorable response to methylphenidate in three cases and spontaneous resolution in one case.

**Conclusion:**

We highlight the importance of identifying cases of post-COVID central hypersomnia, as it may be a treatable trait of a post-COVID condition.

## 1 Introduction

Fatigue is the most commonly reported symptom of post-COVID condition (PCC) and affects more than 40% of all patients, with repercussions in work and daily life several months after infection (1, 2). With several 100 million people infected worldwide, post-COVID fatigue has a significant impact on physical and psychological health of many individuals with important social and economic consequences. Unfortunately, there is still no pharmaceutical treatment for this condition. Post-COVID central hypersomnia is rarely reported and only few data on sleep studies are available (3). In contrast to fatigue, treatments are available for central hypersomnia, in particular when they meet the diagnostic criteria for idiopathic hypersomnia and narcolepsy. We present the clinical history and detailed sleep studies of four patients with proven central hypersomnia triggered by SARS-CoV-2 infection (three idiopathic hypersomnia and one with type II narcolepsy), including the evolution under medication. We then discuss the question of whether SARS-CoV-2 could be added to the viruses possibly involved in the still unclear pathogenesis of idiopathic hypersomnia.

## 2 Methods

From May 2021 to January 2023, 530 patients were referred for evaluation to our post-COVID clinic, which covers a population of around 300,000 inhabitants. Patients complaining of excessive sleepiness were first assessed and treated for a psychiatric disorder, sleep insufficiency, or sedative medications. If sleepiness persisted, they were offered a sleep study, consisting of a polygraphy if there was a high probability of sleep apnea syndrome, if not a polysomnography (PSG). If the mean daily total sleep time estimated by sleep diary was more than 11 h, a 48 h sleep laboratory assessment was proposed, consisting of a 24 h *ad libitum* sleep PSG, a second night PSG, and a multiple sleep latency test (MSLT). If the total sleep time was <11 h, but the daytime sleepiness was judged severe enough to evoke narcolepsy, we performed a PSG and a MSLT.

**Figure 1:**
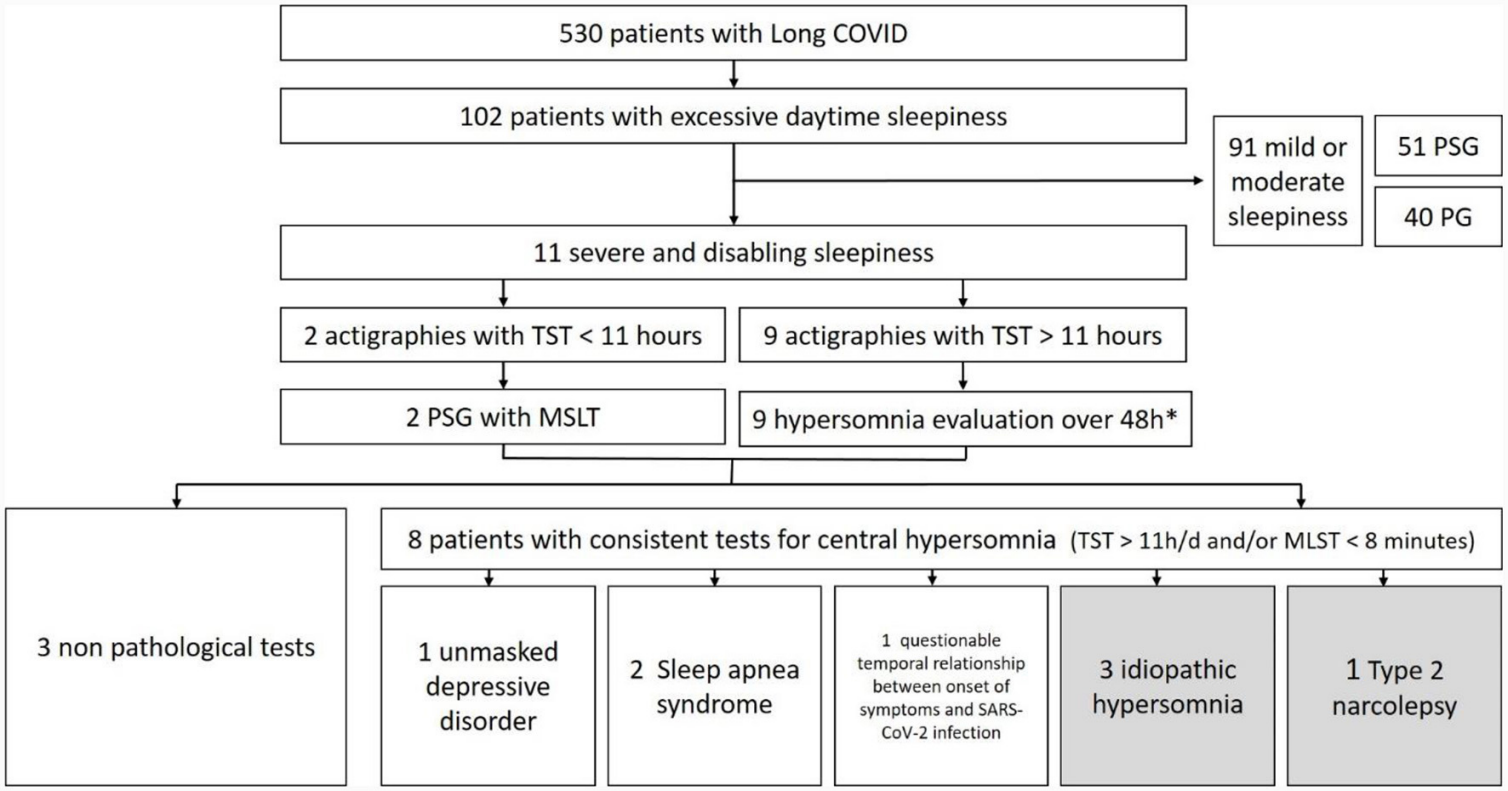
Flow chart of diagnostic trajectories. PSG, polysomnography; PG, polygraphy; TST, total sleep time; MSLT, multiple sleep latency test. *See text for details.

By systematically following this diagnostic process (Figure 1), we identified eight patients with objective central hypersomnia. In order to select only cases formally confirmed as due to SARS-CoV-2 infection, we excluded four cases due to potential confounding factors (two sleep apnea syndromes, one reclassified as a depressive disorder, and one with a questionable temporal relationship between symptom onset and SARS-CoV-2 infection). The remaining four patients linked the onset of sleepiness to their SARS-CoV-2 infection, which were all confirmed by polymerase chain reaction testing and treated in an ambulatory care unit. Of these, three met the International Classification of Sleep Disorders Third Edition (ICSD-3) criteria for idiopathic hypersomnia and one for type II narcolepsy. Drug, psychoactive medication, sleep insufficiency, a psychiatric disorder, and other causes of sleepiness were ruled out by a thorough anamnesis, physical assessment, actigraphy, biological workup, PSG, and brain nuclear magnetic resonance imaging. All patients gave informed and written consent to the present publication.

## 3 Case descriptions

### 3.1 Patient 1

Patient 1 was a previously healthy 18-year-old male student who presented a SARS-CoV-2 infection in November 2020. Before COVID-19, his usual sleep duration was about 8–9 h per day. After the infection, he experienced excessive daytime sleepiness and a significant increase in his sleep requirements, up to 12–14 h per day. Five months later, he was still unable to return to school due to difficulty wakening up for early morning classes, as well as falling asleep in class. The 48 h sleep studies and tests performed 10 months after the initial SARS-CoV-2 infection are reported in Table 1. The diagnosis of idiopathic hypersomnia was made according to ICSD-3 criteria. A sleep onset rapid eye movement period (SOREMP) was observed at the 9 a.m. nap, which is common at this age.

**Table 1:** Clinical data and results of sleep studies and sleep latency tests.

	**Case 1**	**Case 2**	**Case 3**	**Case 4**
Gender and age (years)	M, 18	F, 38	M, 54	F, 18
BMI, kg/m^2^	19.0	23.7	22.6	20.3
Time from acute COVID, weeks	45	40	76	108
Epworth sleepiness scale	21	11	11	15
HADS	11	17	11	10
SF-36	95	92	106	102
Sleep onset latency, min	3.8	9.7	0.5	6.9
WASO, min	38.1	13.5	93.5	15
Sleep efficiency, %	92	98	82	98
Slow-wave sleep, %	22	22	21	19
REM sleep, %	14	27	28	17
Micro-arousals index,/h	20	17	17	8
AHI/h	0.6	1.8	4.7	0.5
RERA/h	2.2	2.8	4.7	0.7
ODI,/h	0.1	1.2	3.7	0.1
Mean SpO2, %	95.0	94.9	95.5	95.3
PLM index/h	1	9	4	0
TST over 24 h *ad libitum* sleep, h:min	12:26	10:28	NA	14:32
Mean sleep latency on 5 naps-MSLT, min	7.2	8.1	7.7	8.2
SOREMP, *n*	1	0	3	0

Polysomnography results refer to the first night of the 48 h workup. M, male; F, female; BMI, body mass index; HADS, hospital anxiety and depression scale; SF-36, 36-item short form health survey; WASO, wakefulness after sleep onset; AHI, apnea hypopnea index; RERA, respiratory effort-related arousal; ODI, oxygen desaturation index; PLM, periodic limb movement; NA, not available; TST, total sleep time; MLST, multiple sleep latency test; SOREMP, sleep onset rapid eye movement period.

As the attentional deficit related to sleepiness had a major impact on the patient, he was prescribed methylphenidate 40 mg/d, which was effective on sleepiness, fatigue, and concentration in class. His sleep requirements decreased back to 8–9 h/day and he was able to return to school with complete resolution of drowsiness, lateness and absences. A trial to stop methylphenidate failed after 4 months (13 months after his infection) with a resurgence of hypersomnia (sleep duration more than 11 h per day). Three years after the infection, he continues taking this medication.

### 3.2 Patient 2

Patient 2 was a previously healthy 38-year-old female who suffered from a SARS-CoV-2 infection in October 2020. Before COVID-19, her usual sleep duration was about 9 h per day. Approximately 4–6 weeks after infection, she developed excessive daytime sleepiness and increased sleep requirements, resulting in absences from work. Eight months after the infection, she still had major difficulties concentrating and an irresistible need for afternoon naps. An actigraphy documented a mean nocturnal sleep time of over 11 h per night on weekdays and about 14 h per night on weekends. Due to the major disability related to sleepiness and actigraphy data, we started methylphenidate at that point. With 20 mg/d, she was able to return to work.

A 48 h sleep laboratory workup was performed about 1 month later, i.e., 9 months after infection. She stopped methylphenidate 5 days before the test to enable interpretation. Results are shown in Table 1. The diagnosis of idiopathic hypersomnia was made according to ICSD-3 criteria on the basis of the symptoms and the aforementioned actigraphy. The borderline MSLT results (mean sleep latency 8.1 min) were attributed to the finally favorable evolution. Indeed, we were able to taper the methylphenidate to 10 mg/d just after the sleep tests and stop it gradually after 2 months with no recurrence of hypersomnia.

### 3.3 Patient 3

Patient 3 was a 54-year-old male treated for hypertension and sleep apnea syndrome for several years who was referred to our clinic by his general practitioner. A SARS-CoV-2 pneumonia was diagnosed in May 2021, but did not require hospitalization. Two months after infection, he was suffering from dyspnea and irrepressible bouts of sleepiness leading to daytime naps. He reported an increase in nocturnal sleep duration from 6 to 7 h per day before SARS-CoV-2 infection to 9–10 h per day afterwards, in addition to one or two naps during the day. Naps were restorative before the rapid reappearance of sleepiness. He also described dizziness, memory, and attention disorders and irritability. He reported no sleep hallucination, sleep paralysis, or cataplexy. There was no evidence of narcolepsy before COVID-19. Continuous positive airway pressure (CPAP) adherence was good (6 h 45 min/night before infection) and effective (no residual fatigue; apnea-hypopnea index, 3.4/h according to built-in software).

The work-up included a cardiopulmonary exercise test that found dysfunctional breathing with hyperventilation. A dedicated respiratory rehabilitation program improved dyspnea. However, attacks of daytime sleepiness persisted and led to a 48 h sleep laboratory workup 17 months after infection (Table 1). These examinations confirmed the effective treatment of sleep apnea syndrome by CPAP. The MLST found a mean sleep latency of 7 min 42 s with three sleep onset rapid eye movement periods, leading to the diagnosis of type II narcolepsy. We proposed modafinil to the patient, which he declined as he felt a slow trend toward the spontaneous improvement of symptoms and was reluctant take any psychotropic medication. He confirmed an improvement 3 months later (20 months after SARS-CoV-2 infection) and we stopped the follow-up.

### 3.4 Patient 4

Patient 4 was an 18-year-old female student with a recent history of infectious mononucleosis in July 2020, with full remission after 1 month. Following her first SARS-CoV-2 infection in November 2020, she gradually developed fatigue with excessive daytime sleepiness and increased sleep requirements. She barely managed to maintain her studies, but sleepiness gradually improved over the year 2021. In January 2022, she presented with a second SARS-CoV-2 infection, which led to a significant recurrence of hypersomnia, including an irrepressible need to sleep in front of her schoolmates. The results of the 48 h sleep laboratory assessment performed in November 2022 are reported in Table 1. We retained the diagnosis of idiopathic hypersomnia according to ICSD-3 criteria. We prescribed methylphenidate 10 mg/three times daily, which is the first-line reimbursed treatment in Switzerland. She takes it only on days when her work requires more concentration (about 3 times/week), with a good effect on sleepiness.

## 4 Discussion

Among 530 patients living with PCC, we identified four cases of objective central hypersomnia. Of these, three met ICSD-3 criteria for idiopathic hypersomnia and one for type II narcolepsy, whereas these pathologies are excessively rare in the general population. The clear temporal link between the onset of symptoms and SARS-CoV-2 infection, together with the exclusion of other causes of central hypersomnia, strongly suggests a causal link between infection and central hypersomnia in these four cases, which could be named “post-COVID central hypersomnia.”

We prescribed methylphenidate in three of the four patients described, which had a very positive effect on excessive daytime sleepiness and daytime functioning. Methylphenidate is generally recommended as a second-line treatment in idiopathic hypersomnia, modafinil being the first line. However, in Switzerland, reimbursement of modafinil is only possible after failure of methylphenidate. Furthermore, as methylphenidate not only has a dopaminergic, but also an adrenergic effect, we postulated that its effect would be broader than modafinil on the cognitive dysfunctions associated with Long COVID. Indeed, these dysfunctions go beyond the wakefulness regulation system and also concern the functions of attention, working memory and cognitive flexibility, which are improved by increased levels of norepinephrine.

The prevalence of central hypersomnia in PCC is probably underestimated in our cohort as we did not report cases of hypersomnia with a more rapid evolution, which resolved before the somnological assessment could be carried out. In addition, we excluded four patients with a potentially questionable link to the infection, even though these patients were profoundly convinced of the responsibility of their viral infection. Regarding the prevalence of post-COVID central hypersomnia in the general population, a minimal prevalence of 1.3/100,000 inhabitants could be suggested as the population covered by our clinic is around 300,000, but it should be emphasized that our study design is not adequate for determining a prevalence, which is obviously subject to numerous biases.

Central hypersomnia may be one of the many neurological impairments described in PCC, whose pathophysiology remains unclear (1, 3). Several hypotheses have been proposed, including neurological damage, immune system dysregulation, autonomic nervous system dysfunction and a persistent viral presence (4). These mechanisms could explain a prolonged dysfunction of brainstem nuclei involved in sleep-wakefulness regulation (5). Although no routinely available paraclinical test can currently confirm the diagnosis of PCC, certain non-specific neuroimaging changes have been reported (6).

According to our observations, SARS-CoV-2 could be added to the viruses possibly involved in the still unclear pathogenesis of the so-called “idiopathic” hypersomnia, such as Epstein Barr virus and SARS-CoV-1 (7, 8). Moldofsky and Patcar (7) showed an association between myalgic encephalomyelitis/chronic fatigue syndrome (ME/CFS) and SARS-CoV-1 infection, with five of 22 patients showing excessive sleepiness confirmed by MLST. To date, two cases of narcolepsy triggered by SARS-CoV-2 infection have also been described (9, 10). Viral components used in vaccines may also trigger central hypersomnia, similar to the increased risk of narcolepsy shown with H1N1 vaccination (10), or the reported recurrence of severe hypersomnia after a SARS-CoV-2 vaccine in a patient previously treated for post-Epstein-Barr virus hypersomnia (11). Similarly, SARS-CoV-2 infection triggered an exacerbation of Kleine-Levin syndrome in 2 cases (12, 13). These data suggest a potential common immunological action of different viruses on the function of brainstem nuclei involved in sleep-wakefulness regulation.

Retornaz et al. (14) found high clinical and biological similarities between long COVID and ME/CFS. However, central hypersomnia is a clearly distinct entity as excessive sleepiness is not a classical symptom of ME/CFS. This was illustrated by Neu et al. (15) who reported MSLT in 16 ME/CFS patients, with a MSLT within the norm.

Our study has some limitations. First, the causal link between SARS-CoV2 infection and hypersomnia in our four cases is not formally proven. A causal link is certainly strongly suggested by the clear temporal relationship and the rigorous exclusion of other causes of hypersomnia, but there is currently no test that can formally attribute a symptom to PCC. Second, the prevalence of post-COVID central hypersomnia cannot be precisely estimated by our data as discussed above. Third, we only have one sleep laboratory assessment per case that was performed quite late after the onset of symptoms, which does not allow to describe the objective evolution of hypersomnia over time. Finally, the small number of cases limits the conclusions that can be drawn about the treatment and evolution of this condition. Although there was a clear improvement in sleepiness in two of four cases at around 12 and 20 months from symptom onset, this is insufficient to draw any general conclusions about the course of post-COVID central hypersomnia. Further research is therefore still needed in this area.

## 5 Conclusion

Fatigue is the most frequent symptom in patients with a PCC, yet without any effective treatment. We report four cases of post-COVID central hypersomnia, with a favorable response to methylphenidate in three patients. Therefore, it is important to identify central hypersomnia in these patients as it may be a treatable trait of long COVID. In addition, post-COVID central hypersomnia could also serve as a model to better understand post-infectious hypersomnia.

## Data availability statement

The original contributions presented in the study are included in the article/supplementary material, further inquiries can be directed to the corresponding author.

## Ethics statement

Ethical review and approval was not required for the study on human participants in accordance with the local legislation and institutional requirements. The studies were conducted in accordance with the local legislation and institutional requirements. The participants provided their written informed consent to participate in this study. Written informed consent was obtained from the individual(s) for the publication of any potentially identifiable images or data included in this article.

## Author contributions

CM-Z: Conceptualization, Data curation, Investigation, Writing – original draft, Writing – review & editing. AV-C: Data curation, Formal analysis, Methodology, Writing – review & editing. NC: Writing – review & editing. LG: Writing – review & editing. MA: Writing – review & editing. AB: Writing – review & editing. IF: Writing – review & editing. P-OB: Methodology, Supervision, Writing – review & editing. GG: Conceptualization, Methodology, Supervision, Validation, Writing – review & editing.
